# How Many Single Rulebooks? The EU’s Patchwork Approach to Ensuring Regulatory Consistency in the Area of Investment Management

**DOI:** 10.1007/s40804-021-00228-w

**Published:** 2022-02-24

**Authors:** Kian Navid

**Affiliations:** Maastricht University, Maastricht, The Netherlands

**Keywords:** Regulatory consistency, Financial regulation, EU Single Rulebook, UCITS, AIFMD, Investment management, Financial crisis

## Abstract

The principle of consistency has undergone a remarkable evolution in the European Union (EU) from a mere political objective to a justiciable constitutional principle of EU law. In the area of financial regulation, regulatory consistency plays a particularly salient role as it is considered a prerequisite for preserving financial stability. In the wake of the 2007/2008 financial crisis, EU policy makers coined the concept of the Single Rulebook, highlighting the importance of a consistent and unified regulatory framework for the EU financial sector with a view to completing the Single Market in financial services and ensuring financial stability. This article examines the progress made towards achieving the Single Rulebook in the area of investment management over a decade after the financial crisis. The post-crisis EU legislation in this area follows a patchwork approach with a multitude of Level 1 and Level 2 directives and regulations that largely rely on the contrived legal form and labels to determine the applicable rules. This form-over-substance approach has created a complicated regulatory regime that is often detached from actual risks for investors and financial stability and thus fails to achieve the overarching policy goal of ensuring regulatory consistency. The central argument put forward in this article is that the Single Rulebook would be better achieved with a substance-over-form approach that addresses substantially similar investor protection and financial stability risks in a consistent manner.

## Introduction

Since the 2007–2008 financial crisis there has been a broad consensus that the regulation and supervision of the financial sector need to be fundamentally overhauled. To address perceived shortcomings in the pre-crisis policies, the regulatory reform agenda introduced a broad range of legislative measures aimed at increased investor protection and financial stability. Both internationally at the G20 level and at the level of the European Union (EU), the lack of regulatory consistency was identified as one of the major deficiencies of the pre-crisis policies and thus the need to ensure the consistency of the post-crisis regulatory framework has been moving increasingly into the spotlight.^1^ In the EU, the endeavour to achieve regulatory consistency in the area of financial regulation has even prompted the creation of the EU Single Rulebook concept which signifies the need to build a unified and consistent set of rules for the EU Single Market. While the various individual pieces of financial legislation that were adopted over the past decade have been subject to much analysis and debate in the literature,^2^ the question of how consistent the EU post-crisis regulatory framework actually turned out to be still remains underinvestigated.

This article focuses on the role of regulatory consistency in EU law and examines the progress achieved towards the Single Rulebook in the area of investment management. The investment management sector is of a vital importance for the EU economy and plays a significant role in global capital markets. In terms of economic salience, the combined 17.1 trillion EUR managed by regulated EU investment managers^3^ exceed the GDP of the entire EU which for 2019 was estimated at 13.9 trillion EUR.^4^ These figures are without even accounting for the 1.7 trillion EUR of net assets in investment funds marketed by non-EU fund managers in the EU, which are also partly subject to EU regulation.^5^

The findings of this analysis indicate that the post-crisis EU reform agenda ended up building a rather complex patchwork regime with a multitude of individual directives and regulations that ultimately follow a form-over-substance approach, meaning that the applicable rules are not based on actual asset or product-specific risks but on the contrived legal form and label of the product as well as the regulatory status and location of its manager. This paper therefore suggests that a true Single Rulebook would be better achieved with a single directly applicable regulation that follows a risk-based substance-over-form^6^ approach. This requires that substantially similar risks posed by financial market participants and financial products are treated in a consistent manner unless the asset and product-specific risks posed by them for investors or the stability of the financial system would justify different regulatory treatment.

This article is structured in six sections. Section 2 provides a basis for understanding the importance and position of the principle of consistency in EU law and beyond. Section 3 reflects on the concept of consistency and clarifies the use of terminology in this article. Section 4 elaborates on the elevated significance that is assigned to the principle of consistency in the area of EU financial regulation. Subsequently, Section 5 of this article examines the consistency of EU financial regulation in the area of investment management and thereby sheds light on the question of to what extent the EU has been able to meet its policy goal of creating a consistent and unified rulebook for financial markets. Finally, Section 6 provides a set of conclusions.

## Understanding the Importance of Consistency in EU Law

The effort to achieve consistency in financial regulation is founded on the principle of consistency which has undergone a remarkable evolution at the level of the EU over the past decades. Although consistency was not explicitly mentioned in the Treaty of Rome, it has been argued that the foundations for the principle of consistency were present^7^ since it obliged all Member States to abstain from any measure which could jeopardise the attainment of the objectives of this treaty.^8^ The first explicit references to the principle of consistency at the treaty level were made over three decades ago, aiming to coordinate the conduct of the various actors involved in the European Community (EC) in the area of external relations.^9^ It was therefore a rather limited principle in terms of its scope and was to be considered a mere political standard within the EC legal system without the European Court of Justice’s direct jurisdiction.^10^

The Treaty of Maastricht reinforced the importance of the principle of consistency since it required, *inter alia*, that the EU is served by a single institutional framework which shall ensure the consistency and the continuity of the activities carried out in order to attain its objectives while respecting and building upon the *acquis communautaire*.^11^ The principle of consistency was thus recognised as a constitutional principle of the EU’s legal order.^12^ The Maastricht Treaty emphasised in particular the consistency of external activities as a whole in the context of the Union’s external relations, security, economic and development policies.^13^ Moreover, it specifically designated the Council and the Commission as being responsible for ensuring such consistency.^14^

The Treaty of Lisbon gave even greater attention to the role of consistency as it included numerous references thereto in both the Treaty on European Union (TEU) and the Treaty on the Functioning of the European Union (TFEU) as a legal obligation assigned to EU institutions.^15^ These Treaties notably require that the Union shall have an institutional framework which shall ensure the consistency, effectiveness and continuity of its policies and actions^16^ and set out that the Union shall ensure consistency between its policies and activities.^17^ The broad wording of the new provisions encompasses not only all of the EU institutions, but also extends to all other EU actors such as the agencies and is no longer limited to ensuring consistency between external EU policies alone.^18^ In addition, the Treaties provide for a number of specific mandates and institutional competencies for ensuring consistency.^19^ By way of an example, the General Affairs Council shall ensure consistency in the work of the different Council configurations^20^ and the President of the Commission shall decide on the internal organisation of the Commission, thereby ensuring that it acts consistently, efficiently and as a collegiate body.^21^

Moreover, the Lisbon Treaty provided the European Court of Justice with jurisdiction over the consistency principle^22^ and consistency even plays a decisive role for the competencies of EU courts. Decisions given by the General Court may exceptionally be subject to review by the Court of Justice where there is a serious risk of the unity or consistency of Union law being affected^23^ and the General Court may in fact itself consider that a case requires a decision of principle likely to affect the unity or consistency of Union law and refer the case to the Court of Justice for a ruling.^24^ Equally, the Advocate General may propose that the Court of Justice reviews the decision of the General Court, where it considers that there is a serious risk of the unity or consistency of Union law being affected.^25^ This flows from understanding that the consistency of judicial output is a prerequisite for legal certainty and therefore the rule of law. In this respect, the European Court of Human Rights has made it clear that it is the role of a supreme court to ensure the consistency of judicial output.^26^

The principle of consistency is, however, not a unique feature of the EU legal order and is directly or indirectly reflected in many national legal doctrines and constitutions. By way of an example, the common law applies the *Stare Decisis* doctrine (a Latin term meaning ‘to stand by that which is decided’) that obligates the courts to follow historical cases when making a ruling on a similar case. *Stare Decisis* therefore ultimately aims to ensure that cases relating to similar circumstances and facts are approached in a consistent manner in order to avoid that court decisions ‘vary like the Chancellor’s foot’.^27^ An inconsistent decision without an explanation as to why differences are justified is therefore quashed on the grounds that it is irrational and arbitrary.

This endeavour to achieve consistency may in fact be very much explained by neuroscience and social psychology. Cognitive neurobiology recognises the principle of consistency and has shown—both in humans and (other) animals—that when two or more simultaneously active cognitive structures are logically inconsistent, arousal is increased, which activates processes with the expected consequence of increasing consistency and decreasing arousal.^28^ Increased arousal is experienced as aversive, while the expected or actual decrease in arousal as a result of increased consistency is experienced as rewarding.^29^ The principle of consistency has been applied most prominently in social psychology where there is a long and continuing strand of literature^30^ showing that human behaviour is motivated by the desire to eliminate inconsistency between a person and his or her social environment. Most notably, the theory of cognitive dissonance gained wide-ranging prominence.^31^ According to this theory, when two actions or ideas are not consistent with each other, humans make all efforts to change them until they become consistent. The discomfort is caused by people’s beliefs clashing with new information being perceived, wherein they try to find a way to resolve the inconsistency to reduce their discomfort, either by adding new parts to the cognition causing the dissonance or by avoiding circumstances and contradictory information that would likely increase the magnitude of the cognitive dissonance.^32^ Experiments show that people therefore do not only prefer information confirming their own preferences, but they also positively evaluate others who communicate this information and provide them with positive feedback for doing so.^33^

It does not therefore come as a surprise that consistency is a frequently used concept in many official documents and public speeches by EU policy makers,^34^ the wording of which often emphasises the great expectations associated with consistency such as effectiveness, legitimacy and credibility.^35^ In fact, even the wording of the TEU itself repeatedly juxtaposes the notions of consistency and effectiveness or efficiency.^36^

In sum, achieving consistency in financial regulation is not a mere political ambition but is founded on a justiciable constitutional principle that is ultimately based on the rule of law.^37^ The effort to achieve consistency is in fact deeply rooted in human neurobiology and social psychology which explains why consistency is often associated with positive connotations such as effectiveness, legitimacy and credibility.

## Consistency: Defining and Operationalising an Elusive Concept

Despite its relatively long history and increasing importance in EU law, in particular in the area of financial regulation, the concept of consistency remains largely elusive due to a lack of definitions in official documents and the seemingly interchangeable use of the words *consistency* and *coherence* which has been subject to lengthy debate in academic literature. While the English versions of the EU Treaties use the term consistency, the German (‘Kohärenz’), French (‘cohérence’), Italian (‘coerenza’), Spanish (‘coherencia’) and Portuguese (‘coerência’) versions use the word coherence.^38^ In fact, the English versions of the EU Treaties use both the notions of consistency and coherence seemingly interchangeably. Against this background, one strand of literature argues that the debate about consistency versus coherence is like quibbling over semantics and that both terms can be used more or less interchangeably.^39^ Nuttall for example states that ‘they are frequently interchangeable in the texts, and attempts to distinguish between them risk ending in linguistic pedantry’, although he acknowledged that coherence might have a broader signification than consistency.^40^

Another strand of literature focuses precisely on the potentially broader signification of coherence and therefore emphasises the differences between the two notions^41^ whereby consistency usually encapsulates logical compatibility and the absence of contradictions within and across policies and actions, whereas coherence relates more to positive synergies between different fields and actors and added value. In this context, proponents of this view have also pointed at two cases, involving enforcement proceedings against Germany and Luxembourg respectively, where the Court of Justice underlined the need for the Member States to cooperate with the Community institutions to ensure ‘the coherence and consistency of the action’.^42^ While indeed the use of both notions by the Court may signify that they are to be understood as separate concepts, it is also worth noting that the Court did not further specify or distinguish between them, but simply used both terms which may arguably equally support the view that a distinction between the two notions may not be as relevant in practice as it is in the academic debate.

For the sake of clarity, this article uses the notion of consistency given its use in the English versions of the Treaties. Policy consistency is understood in the same way as advocated by the OECD, namely as policies that are not internally contradictory and do not conflict with attaining a given policy objective.^43^

The academic literature also delineates between different dimensions of consistency, in particular vertical, horizontal and institutional consistency.^44^ Horizontal consistency is achieved where there are no contradictions within and across EU policies or actions, whereas vertical (or inter-level) inconsistency arises in case of conflicts between the policies or actions of the EU and its Member States. Moreover, institutional inconsistency refers to inter- and intra-institutional contradictions at the EU level. For reasons of practical delimitation, this article primarily focuses on the horizontal dimension of consistency by investigating whether EU legislation in the area of investment management include internal contradictions that conflict with the overarching policy goals in this area. Nevertheless, some of the findings of this article indicate that the vertical and institutional dimensions of consistency in this area would equally merit more research.

## The Increasing Importance of Consistency in the Area of EU Financial Regulation

After the global financial crisis of 2008, regulatory consistency gained traction both at the international and at the EU level. At the 2008 G20 summit in Washington, world political leaders concluded that in order to achieve financial stability all financial market actors and products must be subject to enhanced regulation and supervision and declared that: ‘We call upon our national and regional regulators to formulate their regulations and other measures in a consistent manner’.^45^ On the whole, the short G20 declaration included over a dozen references to the need for ensuring the consistency of financial regulation. This was followed up with a broad range of commitments made at the 2009 G20 summit in Pittsburgh, including: ‘We are committed to take action at the national and international level to raise standards together so that our national authorities implement global standards consistently in a way that ensures a level playing field and avoids fragmentation of markets, protectionism, and regulatory arbitrage’.^46^

Around the same time, the President of the European Commission, José Manuel Barroso, asked the former governor of the Bank of France and former director of the International Monetary Fund, Jacques de Larosière, to set up a High-Level Group composed of internationally recognised independent experts. This High-Level Group would also be advised by diverse prominent personalities and representatives of European institutions such as Mario Draghi, Jean-Claude Trichet and Baron Alexander Lamfalussy on the future design of EU financial markets regulation and supervision. The highly anticipated report was published in February 2009^47^ and included a number of explicit recommendations and over two dozen references to the need for achieving consistency^48^ in the regulatory framework and supervisory practices in the EU.

For this aim, EU policy makers coined the concept of the ‘EU Single Rulebook’. The core idea of the Single Rulebook is to create a unified (‘single’) framework to ensure the consistent regulation and supervision of all market participants across EU Member States. While the term has not been legally defined in any EU directive or regulation, the clarifications provided by the European Commission indicate that the Single Rulebook aims at achieving the consistency of both policies and (supervisory) actions.^49^ This understanding is shared by the European Supervisory Authorities which routinely link the creation of the EU Single Rulebook to the promises of effectiveness,^50^ resilience,^51^ transparency^52^ and efficiency.^53^

With a view to completing the Single Rulebook and meeting the G20 commitments, EU policy makers adopted a vast number of directives and regulations that aimed at addressing market failures and perceived shortcomings in pre-crisis policies. In this context, it has been estimated that more than 50,000 pieces of financial legislation were published across the G20 between 2009 and 2012 and that over 50,000 regulatory updates were made in 2015 with the revised Markets in Financial Instruments Directive (MiFID II)^54^ in the EU alone having more than 30,000 pages and 1.5 million paragraphs.^55^

Nevertheless, academic literature examining the consistency of EU financial regulation and, more specifically, the actual progress made towards completing the Single Rulebook remains relatively sparse. Literature in the area of EU banking regulation indicates that the completion of an EU Single Rulebook has not yet been accomplished.^56^ In the area of investment management, it has been shown that despite the common driving forces of hedge fund regulation across the Atlantic and the G20 commitments to ensuring consistent regulation, ultimate policy outcomes in the USA and the EU are significantly divergent as EU regulators prioritised EU passport mechanisms which engendered the demand for investor protection while the main concern in the USA was to address the potential systemic risk of hedge funds.^57^

Undertakings for collective investment in transferable securities (UCITS) account for approximately 60% of the overall net assets in the EU investment fund market^58^ and have been subject to financial regulation at the EU level since the mid-1980s, when the first iteration of the UCITS Directive was adopted. To qualify for the UCITS label, the fund must be EU-based and it must be managed by a fund manager that is established in the EU and authorised in accordance with the UCITS Directive.

In contrast, EU legislation did not regulate the other 40% of the investment fund market, namely the broad range of non-UCITS funds such as hedge funds, private equity, venture capital, real estate, infrastructure or commodity funds, until more recent years. Following the financial crisis, public perception of this so-called alternative investment sector started to change and many politicians and regulators argued that a comprehensive and consistent regulatory framework was needed. Due to the financial crisis, the regulation of this sector, notably the regulation of hedge fund and private equity managers, received extensive attention from politicians and the media which caused the European Commission to publish, in April 2009, a proposal for a Directive for Alternative Investment Fund Managers (AIFM Directive).^59^ This Directive primarily aims at extending appropriate regulation and supervision to the alternative investment fund management sector in the EU Member States. The legal text was already agreed upon in October 2010 by the EU legislators; it then came into force on 21 July 2011 and Member States needed to transpose the framework into national law by 22 July 2013. In addition to the UCITS Directive^60^ and AIFMD^61^ as the main regulatory frameworks on investment funds with numerous implementing and delegated acts, EU legislators also adopted various additional pieces of product and/or manager legislation such as on European Venture Capital Funds (EuVECA),^62^ European Social Entrepreneurship Funds (EuSEF),^63^ European Long-Term Investment Funds (ELTIF)^64^ and Money Market Funds (MMF).^65^ However, only the regulation on MMF is mandatory in the case where funds qualify as MMF, whereas the application of the other three regulations is merely voluntary where market participants would like to use the EuVECA, EuSEF or ELTIF labels for marketing purposes.

The multitude of Level 1 and Level 2 directives and regulations in the area of investment management each regulate certain categories of managers and/or products. Importantly, the relevant rules are not based on specific investment strategies, business activities or actual investor base, but solely on the legal form or label used for the relevant funds. This form-over-substance patchwork approach has significantly increased the operational and regulatory complexity in this area for market participants, competent authorities as well as investors and it raises the question of whether the EU legislators might have inadvertently created several separate and inconsistent rulebooks instead of the ‘Single’ Rulebook that they originally intended to create.

## Inconsistent Regulatory Treatment of Risks Within the EU Single Rulebook

In this section, we study whether the patchwork approach to the EU Single Rulebook amounts to inconsistent regulatory treatment and the implications of this approach from the point of view of the actual risks posed for investors and financial stability. In order to conduct the empirical analysis, it is necessary to examine how the EU legislation regulates different types of investment fund portfolios in practice. Therefore, the applicable EU rules are applied to four investment portfolios (Portfolios A to D) relating to the key asset classes (listed securities, unlisted securities, real assets and derivatives) to shed light on the practical differences in regulatory treatment and to analyse the potentially resulting regulatory inconsistencies. In addition, Portfolio E relates to the scenario where a non-EU manager manages and markets Portfolios A to D to EU investors and therefore illustrates the different regulatory treatment between EU and non-EU managers performing the same activity. The case study is based on the analysis of the relevant EU legislation and accompanying documents such as the Impact Assessments carried out by the European Commission as well as the records of the Council and parliamentary discussions in the course of the legislative initiatives (Table 1).

**Table 1:** Overview of investment portfolios studied

Portfolio A Listed securities	Portfolio B Unlisted securities	Portfolio C Real assets	Portfolio D Derivatives	Portfolio E Non-EU
Investment portfolios composed of listed shares and bonds	investment portfolios composed of unlisted shares and bonds	investment portfolios composed of real (physical) assets such as residential or commercial properties, commodities, infrastructure, wine, art, classic cars etc.	investment portfolios composed of futures, options or more complex derivative instruments relating to securities and financial indices and potentially employing high synthetic leverage	investment portfolios A to D set up outside of the EU and managed by a non-EU manager selling them to investors in the EU

Portfolio A would be commonly referred to as ‘plain vanilla’ funds and many would intuitively associate those portfolios with the terms ‘UCITS’ or ‘mutual funds’.^66^ However, Portfolio A could also be set up as an alternative investment fund (AIF). In fact, AIFs invested in transferable securities represent the most dominant AIF segment.^67^

In contrast, Portfolios B and C would be clearly classified as AIFs since the eligible assets rules set out in the UCITS Directive do not allow for exposures to those asset classes.^68^ Depending on the predominant asset class invested in, those portfolios would be commonly referred to as real estate, infrastructure, commodity, private equity or, where invested in start-up companies, venture capital funds.

Portfolio D would be associated by some people with hedge funds,^69^ whereas industry experts would also be familiar with the terms ‘Alternative UCITS’ or ‘Newcits’. The latter notions are not used in EU legislation but are commonly used to refer to UCITS that employ hedge fund-type investment strategies.^70^ The original UCITS Directive did not allow for the use of derivatives at all. However, investments in derivatives are eligible for UCITS^71^ since the UCITS III revision and although the investment restrictions in the UCITS Directive do not allow for financial leverage (as borrowings must be temporary and are limited to 10%), the use of synthetic leverage via derivatives is possible.^72^

Finally, Portfolio E addresses the situation in which any (or all) of the Portfolios A to D would be established as non-EU funds managed by a non-EU manager and sold to EU investors. This serves the purpose of illustrating the different regulatory treatment based on the location of the fund and the manager.

While the analysis of the EU rules that apply to investment Portfolios A to E reveals substantial regulatory inconsistencies in different areas, four core issues have been identified. For the sake of a focused analysis, the issue selection captures the main substantive divergences between the applicable regulatory frameworks and is representative of the types of inconsistencies observed in other issues as well. These four issues are:

- Authorisation (Sect. 5.1).
- Marketing (Sect. 5.2).
- Risk management (Sect. 5.3).
- Supervisory powers and responsibilities (Sect. 5.4).

The following sections will assess whether the investment Portfolios A to E are subject to consistent EU regulatory requirements in these four areas. Where significantly divergent rules are identified, a further analysis of the differences will be carried out from the point of view of investor protection and financial stability risks.

Two important caveats apply. Firstly, the analysis focuses primarily on key inconsistencies between the Level 1 regimes.^73^ When comparing the entirety of Level 2 requirements in the AIFMD and UCITS frameworks, a broader range of more technical regulatory inconsistencies as well as differences in the precise legal wording can be identified since the newer AIFMD Level 2 requirements are significantly more granular on many key issues compared to those laid down in the UCITS framework. By way of an example, the delegation rules set out in the AIFMD^74^ and UCITS Directive^75^ are to some extent consistent when solely looking at the Level 1 provisions.^76^ However, the AIFMD rules are complemented by a detailed set of Level 2 provisions,^77^ specifying, *inter alia*, the due diligence obligations, required features of the delegate, effective supervision by competent authorities, objective reasons for delegation, conflicts of interest, consent and notification of sub-delegation and the maximum extent of delegation, whereas the UCITS Level 2 rules remain largely silent on these important issues.^78^

Secondly, this article will not elaborate on historic inconsistencies (starting with the mere fact that EU legislation did not regulate non-UCITS at all until the AIFMD rules became applicable as from July 2013, albeit with some additional transitional periods) and the efforts made by the EU legislators in more recent years to ensure consistency between the two frameworks, notably with the UCITS V revision^79^ that had to be transposed and applied as from 2016 and which specifically aimed at enhancing consistency between the UCITS and AIFMD framework in the areas of manager remuneration, depositary rules and administrative sanctions.^80^ While, indeed, the latest UCITS recast managed to achieve greater consistency with the AIFMD, it also introduced many new regulatory inconsistencies that would go beyond the scope of this article such as, for example, the introduction of a whistleblower protection regime under UCITS while no such regime was introduced in the AIFMD where whistleblowers remain unprotected without any objective reason for such different legal treatment.^81^

### Authorisation

#### Threshold or no Threshold?

The authorisation regimes in the AIFMD^82^ and UCITS Directive^83^ are largely consistent in terms of the *procedural* requirements for obtaining a licence. However, the regimes differ fundamentally in terms of their *scope of application*. The UCITS Directive covers EU collective investment schemes in transferable securities or in other liquid financial assets and the managers thereof. All UCITS are subject to the UCITS authorisation requirements^84^ and each UCITS manager, irrespective of size or the use of leverage, is obliged to obtain a licence.^85^ Therefore, even a UCITS manager with an unleveraged UCITS portfolio of negligible size would be obliged to seek authorisation under the UCITS Directive and ensure compliance with the full UCITS regulatory framework including the detailed investment restrictions set out therein.^86^

In contrast, the AIFMD only regulates the managers and leaves the regulation of the funds to national legislation. Consequently, the Single Rulebook does not even attempt to ensure the vertical consistency of the product rules and investment restrictions of most AIF categories and therefore tolerates vertical inconsistencies in this key area.^87^ Moreover, the AIFMD follows a threshold-based approach whereby managers of AIFs which manage portfolios of AIFs whose assets under management, including any assets acquired through the use of leverage, in total do not exceed a threshold of 100 million EUR are exempted from the obligation to be authorised and to comply with the full AIFMD regime.^88^ The thresholds are even increased to 500 million EUR when the portfolios of AIFs consist of AIFs that are both unleveraged and have no redemption rights exercisable during a period of five years following the date of initial investment in each AIF.^89^

Importantly, the EU legislators chose to implement the AIFMD as a minimum harmonisation directive, meaning that Member States may impose stricter requirements.^90^ This legislative approach is contrary to the advice given in the de Larosière Report which argued that [emphasis added]

[f]uture legislation should be based, wherever possible, on regulations (which are of direct application). When directives are used, the co-legislator should strive to achieve *maximum harmonisation* of the core issues. Furthermore, a process should be launched to remove key-differences stemming from the derogations, exceptions and vague provisions currently contained in some directives.^91^

As a consequence of this, some Member States have introduced stricter requirements for various aspects of the AIFMD including its scope. By way of an example, the national regime in Portugal^92^ includes additional requirements for sub-threshold managers, whereas Italy^93^ has reduced or effectively removed the thresholds altogether. This means that the necessity to be authorised and to comply with the AIFMD (or similar national requirements) with respect to Portfolios A to D varies significantly depending on the Member State in which the manager is established. Sub-threshold market participants performing the same services would be subject to regulation and supervision in one Member State, while remaining unregulated and unsupervised in another. Therefore, the AIFMD fails to achieve vertical consistency on the salient question of its scope.

In sum, the question of whether fund managers of Portfolios A to D are subject to EU authorisation and regulation at all is treated in a largely inconsistent manner depending on the legal categorisation of the fund as UCITS or AIF as well as on the location of the manager and the relevant national legislation (Table 2). In this context, Member States are permitted to reduce or remove the thresholds altogether or to introduce additional national requirements for managers below the thresholds and the product regulation of AIFs with the applicable investment restrictions even being left entirely to the discretion of national legislation. This legislative approach contrasts starkly with the UCITS framework whereby all managers of UCITS must be established in the EU^94^ and are obliged to seek authorisation and comply with the full UCITS framework irrespective of size or the use of leverage.

**Table 2:** Is the manager obliged to be authorised and to comply with the full AIFMD/UCITS regulatory frameworks?

Portfolio A	Portfolio B	Portfolio C	Portfolio D	Portfolio E
(a) If set up as UCITS: Yes, irrespective of size or use of leverage (b) If set up as AIF: - No if below 100 million EUR - No if below 500 million EUR and no leverage or redemption rights within five years	- No if below 100 million EUR - No if below 500 million EUR and no leverage or redemption rights within five years	- No if below 100 million EUR - No if below 500 million EUR and no leverage or redemption rights within five years	(a) If set up as UCITS: Yes, irrespective of size or use of leverage (b) If set up as AIF: - No if below 100 million EUR - No if below 500 million EUR and no leverage or redemption rights within five years	No, irrespective of size, use of leverage or redemptions rights

The consequences of this form-over-substance approach are even more noticeable for Portfolio A and Portfolio D. Given the eligibility of transferable securities and derivatives for UCITS,^95^ managers of these portfolios could essentially choose whether to opt for the UCITS or AIFMD regimes. In light of horizontal inconsistencies between the UCITS and AIFMD frameworks with the latter adopting a threshold-based approach, this gives rise to an unlevel playing field and regulatory arbitrage risks. Given the vertical inconsistencies due to varying nation approaches to the AIFMD thresholds and product rules, managers of Portfolios A and D may also be inclined to pick a Member State whose national legislation on AIFs would be more favourable. Needless to say that diverging national legislation creates competitive distortions and further increases regulatory arbitrage and unlevel playing field issues which the Single Rulebook is meant to prevent. As a result of this, a number of Member States have recently called on the European Commission to ensure greater consistency in how sub-threshold AIFMs are regulated across the EU.^96^

#### Same Financial Services but Different Rules

With respect to non-EU managers (Portfolio E), it is worth noting that UCITS are by definition EU-based^97^ and must be managed by authorised entities established in the EU.^98^ In contrast, the AIFMD provides for a third country regime and does not prevent non-EU managers from managing EU AIFs or selling non-EU AIFs managed by them to EU investors.^99^ However, it does not yet provide for an authorisation regime for non-EU managers marketing their EU or non-EU AIFs to EU investors despite positive advice by ESMA to the European Parliament, the Council and the Commission on the extension of the AIFMD authorisation and passport regimes to managers from numerous third countries.^100^ In the absence of the third country authorisation and passporting regime, the Single Rulebook leaves this important question to national legislation which further raises the risks of vertical inconsistencies. In fact, the vast majority of Member States do allow non-EU AIFMs to market their funds to investors in their Member State and in many cases non-EU managers are not subject to any additional substantial rules but merely to registration or notification requirements.^101^

As a consequence of this, EU and non-EU market participants performing the same services would be subject to vastly different regulatory treatment. The fact that the application of the full AIFMD regime is subject to the *location* of the manager appears unjustifiable from an investor protection and financial stability point of view. The inconsistent regulatory treatment between AIFs managed by EU and non-EU managers is even more astounding when looking at the economic significance of non-EU AIFs marketed to EU investors. Overall, 900 non-EU AIFs with a total net asset value (NAV) of 1.7 trillion EUR are marketed in 13 EU Member States.^102^ The regulatory Assets under Management (AuM) of these funds is even reported to reach 10.3 trillion EUR, largely due to the high amount of exposure of offshore hedge funds to interest rate derivatives.^103^

In this context, it is also important to note that the form-over-substance approach followed in the AIFMD does not allow for a distinction between the type of assets invested in or the investment strategies employed by non-EU AIFs. As a consequence of this, unleveraged US mutual funds invested in blue chips or US exchange traded funds (ETFs) tracking major financial indices (such as the Dow Jones or S&P500) are tarred with the same regulatory brush as highly leveraged offshore hedge funds invested in illiquid or speculative assets. To this end, one could argue that the AIFMD third country regime actually offers the worst of both worlds since EU and non-EU managers performing the same services are subject to different regulatory treatment without any objective reason, while at the same time it treats all different types of non-EU AIFs with a ‘one size fits all’ approach without any regard to their (different) asset or product-specific risks.

### Marketing

#### Suitability Argument

While the regulatory differences in Sect. 5.1 appear to be inconsistent and even illogical from a financial stability and investor protection risk perspective, the question arises whether they could at least be partially explained from the point of view of potential differences between the types of UCITS and AIF investors and associated investor protection risks (Table 3). This is because the UCITS Directive provides for a marketing passport that encompasses both retail and professional investors, whereas the AIFMD passport only covers the latter. This passporting limitation under the AIFMD is based on the mere assumption that AIFs are more complex and risky products that are more suitable for professional investors, whereas UCITS are less risky and simple products suitable to be sold to the mass market.

**Table 3:** Can the product be marketed to retail investors?

Portfolio A	Portfolio B	Portfolio C	Portfolio D	Portfolio E
(a) If set up as UCITS: Yes, via EU passport (b) If set up as AIF: Only if permitted under the national legislation of the relevant Member State and subject to the requirements set out therein	(a) If set up as EuVECA: Yes, via EU passport (provided investor commits a minimum 100,000 EUR and provides a self-declaration) (b) If set up as AIF: Only if permitted under the national legislation of the relevant Member State and subject to the requirements set out therein	(a) If set up as ELTIF: Yes, via EU passport (subject to additional requirements and a minimum investment of 10,000 EUR) (b) If set up as AIF: Only if permitted under the national legislation of the relevant Member State and subject to the requirements set out therein	(a) If set up as UCITS: Yes, via EU passport (b) If set up as AIF: Only if permitted under the national legislation of the relevant Member State and subject to the requirements set out therein	Only if permitted under the national legislation of the relevant Member State and subject to the requirements set out therein

This simplistic view does not necessarily match reality, however. In terms of complexity, a fund that buys and sells or rents real estate property (Portfolio C) is certainly more easily understood by the average consumer than the alternative UCITS or Newcits with complex derivatives (Portfolio D) that might not even be fully understood by most industry experts. Moreover, claiming that investments in Portfolios A and D are ‘less risky’ than the AIFs Portfolios B and C would raise the question of how such ‘riskiness’ would be measured? This is particularly true since Portfolios A and D could even be set up as AIFs which would further point to the arbitrary nature of the legalistic division between UCITS and AIFs.

This observation is further underlined by the fact that the European Central Bank estimates that the total size of the euro area market for funds pursuing hedge fund strategies amount to 419 billion EUR, out of which 236 billion EUR can be classified as AIFs, and 183 billion EUR of funds can be classified as UCITS. Consequently, in many cases, fund managers use UCITS to offer AIF-style strategies to EU investors including retail investors.^104^ In other words, 44% of all hedge funds sold in the EU are in fact set up as UCITS and could therefore be passported to retail investors across the EU.

Nevertheless, the European Commission introduced the regulatory division between UCITS and AIFs stating that AIFs are not ‘suitable’ for marketing to retail investors, *inter alia*, because of the higher volatility, leverage and liquidity risks of AIFs compared to UCITS as well as lower investment diversification and redemption frequencies.^105^ Interestingly, the Commission did not provide any quantitative data or evidence to back up these assumptions which therefore remain largely unsubstantiated. In fact, recent data indicates that the vast majority of AIFs are unleveraged (85%) and offer at least monthly portfolio and investor liquidity (61%), many of those even offering *daily* liquidity (62%), which indicates that, in the majority of cases, the regulatory division between UCITS and AIFs is not grounded on actual asset or product-specific features and related risks for investors or financial stability, but merely on unsubstantiated assumptions.^106^

More importantly, the alleged lack of ‘suitability’ of AIFs for retail investors does not mean that the relevant managers would be prevented from marketing their AIFs to retail investors because paradoxically the AIFMD, in the end, simply avoided regulating this important question in a consistent manner at the EU level and left it again to national legislation.^107^ Bearing in mind the argument raised by the European Commission that AIFs are not ‘suitable’ for retail investors, the question arises why the Single Rulebook would not regulate the marketing of allegedly ‘unsuitable’ products to EU retail investors? Today, the vast majority of national legislations, in fact, do allow the marketing of AIFs to retail investors without any or relatively light additional requirements (usually in the form of additional national registration and/or disclosures).^108^ Hence, at the very least, one must conclude that the EU legislation has not yet succeeded in ensuring vertical consistency on how this important question is regulated across the EU Single Market.

Indeed, at least^109^ one out of six investors in an AIF today is a retail investor, with the figures being significantly higher for certain types of AIFs.^110^ While there are no official figures for UCITS (and Sect. 5.4 below will elaborate on why this lack of data is to be explained by other regulatory inconsistencies), industry associations reported that 30% of all investors in European investment funds (including both UCITS and AIFs) are retail investors.^111^ The available data therefore indicates that the legalistic division between UCITS as retail products and AIFs as products for professional investors does not match reality since retail investors are significantly exposed to both UCITS and AIFs, while in both cases professional investors are the dominant investor base.

It is also worth noting that the EU legislation allows for a combination of different regulatory licences, meaning that a fund manager may obtain both the licence to act as an UCITS management company and an AIFM.^112^ In such a case, the same manager may, for example, manage two portfolios invested in listed securities (Portfolio A), one of them set up as UCITS and the other (identical one) as AIF. In such a case, the exact same investment portfolio may be subject to the different regulatory regimes including on marketing to (retail) investors, depending merely on the choice of the fund manager to pick the UCITS or AIF label.

Moreover, it is important to note that neither the UCITS Directive nor the AIFMD explicitly distinguish between different types of securities, meaning that, for example, investment portfolios in investment-grade bonds with high credit ratings are essentially treated in the same way as bonds of issuers with low credit worthiness (‘junk bonds’). One of the paradoxical consequences of this form-over-substance approach is that an AIF portfolio invested exclusively in the most creditworthy bond issuers would not benefit from a marketing passport to retail investors, whereas UCITS invested in bonds with low credit ratings would benefit from the EU passport and this is in fact a popular UCITS market segment (albeit using the euphemism ‘*high-yield* bonds’ for marketing purposes instead of ‘junk bonds’).^113^

#### Different Treatment of Venture Capital

With respect to investments in unlisted securities (Portfolio B), it is worth noting that the EU Single Rulebook provides for yet another interesting inconsistency in relation to the marketing rules, namely in the EuVECA Regulation.^114^ This is because the fund managers of venture capital portfolios complying with that regulation would also be able to market to retail investors provided that they invest a minimum of 100,000 EUR and make a self-declaration that they are aware of the risks associated with the investment.^115^ This approach appears to be at odds with the suitability argument made by the European Commission since venture capital (i.e. investments in start-up companies that are in their make-or-break phase) is commonly regarded as one of the riskiest asset classes and therefore riskier than real estate or many other alternative investment strategies where such a marketing passport is not available.

In fact, the European Commission’s Impact Assessment includes numerous references to the high risks involved with venture capital investments, *inter alia*, describing them as a ‘*very risky* type of asset class’.^116^ The Impact Assessment also includes statements that venture capital investments entail a *higher* risk than private equity funds (AIFs) and *lower* returns than private equity funds pursuing a buy-out strategy.^117^ Hence, even if one follows the line of reasoning that it is acceptable to market presumably riskier funds to wealthier retail investors (i.e. those that can commit at least 100,000 EUR), the question arises why this should be limited to EuVECA alone and not extended to all other types of (presumably lower risk) AIFs such as private equity or real estate funds? From an investor protection perspective, this outcome is highly paradoxical bearing in mind also the fact that the EuVECA Regulation is significantly less burdensome compared to the AIFMD regime. In other words, supposedly *riskier* investments are made subject to *lesser* regulatory requirements and even rewarded with an EU-wide passport to market to *retail* investors.

Importantly, the European Commission’s Impact Assessment in 2011 acknowledged that this policy approach does ‘lack coherence’ compared to the general AIFMD marketing regime.^118^ The Impact Assessment therefore concluded that an integration of the EuVECA rules in the AIFMD regime would pose a problem because ‘this lack of legal coherence introduces additional complexity into the already complex provisions of the AIFMD’.^119^ As a result of this, the Commission expressed a preference for the policy option to create a stand-alone regulation for EuVECA. In other words: since the integration of the desired EuVECA rules in a single rulebook for alternative investment funds and their managers would expose legal inconsistencies and add to the overall regulatory complexity, one simply tried to avoid giving too much visibility to this inconsistency by creating yet another label with a separate rulebook (EuVECA Regulation).

Not only does this approach contradict the EU Single Rulebook objective, but the horizontal inconsistencies also appear to be unjustifiable from an investor protection and financial stability perspective. They are, however, easily explained by the EU’s *political* ambition to allocate more capital to small and medium-sized enterprises as part of the Europe 2020 Strategy.^120^ Hence, the inconsistent legal treatment is not based on actual risks for investors or financial stability but seems rather to be motivated by political ambitions and preferences. This indicates that the form-over-substance approach applied in the EU is mainly driven by political considerations. In fact, the European Commission’s legislative proposals on EuVECA^121^ originally included additional safeguards to mitigate these investor protection risks such as the requirement for the EuVECA manager to undertake an assessment of the expertise, experience and knowledge of the investor to seek assurance that all investors are capable of making their own investment decisions and understanding the risks involved and that a commitment of this kind is appropriate for them. However, some Members of the European Parliament expressed a preference for further reducing ‘bureaucratic rules’ and ‘red tape’ to attract more investors to provide investment capital to small and medium-sized enterprises.^122^ The Council even supported an outright deletion of the investor protection safeguards proposed by the Commission pertaining to the assessment of the expertise, experience and knowledge of wealthier retail investors and instead proposed to solely require that those investors present the EuVECA manager with an assessment made by a credit institution, MiFID investment firm or UCITS management company certifying their experience, expertise and knowledge to invest in ‘risk capital’.^123^ Surprisingly, the final political compromise after trilogue negotiations was to reduce the safeguards proposed by (both) the European Commission and Council even further, namely to a mere *self-declaration* that those retail investors are aware of the risks associated with the envisaged investment in EuVECA.

#### Unnecessary Trade-offs

With regard to Portfolio C, another important exception can be observed from the general rule that AIFs cannot be marketed via an EU passport to retail investors. This is because funds invested in long-term investment projects, in particular infrastructure, could be set up as ELTIFs subject to the ELTIF Regulation. Authorised ELTIFs would benefit from an EU passport that also encompasses marketing to retail investors. However, unlike the EuVECA Regulation, the ELTIF Regulation provides for a broad list of investor protection safeguards where funds are marketed to retail investors.^124^ By way of an example, ELTIF investors must benefit from the availability of local facilities in each Member State for the subscription and redemption process and the provision of all required information.^125^ The suitability of the fund for the individual retail investor must be specifically assessed taking into account the knowledge and experience of the investor in the relevant investment field, its financial situation including the possibility to bear losses and its investment objectives and time horizon.^126^ Where the financial instrument portfolio does not exceed 500,000 EUR, retail investors are not permitted to invest an aggregate amount exceeding 10 % of their financial instrument portfolio in ELTIFs and the initial minimum amount invested in one or more ELTIFs must be 10,000 EUR.^127^ Moreover, retail investors shall be provided with appropriate investment advice by the manager of the ELTIF or the distributor before their investment decision^128^ and have the right during the subscription period and at least two weeks after the date of their subscription to cancel their subscription and have the money returned without penalty.^129^

The aforementioned examples only constitute a subset of the additional investor protection safeguards included in the ELTIF Regulation. From a regulatory consistency point of view, the question arises why retail investors in infrastructure portfolios are benefiting from the significantly increased investor protection safeguards compared to investors in venture capital portfolios subject to the EuVECA Regulation. Equally, the question arises why should not all retail investors in AIFs (i.e. beyond ELTIFs and EuVECA) benefit from a consistent level of protection?

In this context, it is interesting to note that the European Commission’s Impact Assessment actually contemplated ensuring horizontal consistency between the EuVECA and ELTIF Regulation as regards marketing to retail investors.^130^ However, the Commission concluded that ‘this approach would not address inconsistencies in *national* rules related to retail funds, and so fragmentation in the market and divergent investor protection standards would be likely to remain’.^131^ In other words, the Commission took the view that ensuring *vertical* consistency in the marketing of ELTIFs to retail investors would be more important than *horizontal* consistency with the EuVECA Regulation. This raises the question of why the European Commission engaged in such a trade-off and what would have prevented it from making legislative proposals that achieve *both* vertical and horizontal consistency? In fact, the Commission revisited the idea to ensure horizontal consistency only a few years after the adoption of the ELTIF Regulation, when revising the EuVECA and EuSEF Regulations in 2016. However, it ultimately concluded that horizontal consistency with the ELTIF Regulation would not be desirable stating that: ‘as the EuVECA and EuSEF are for the time being a niche market, it seems more appropriate to let this market develop with a light touch regime before introducing additional layers of investor protection requirements’.^132^

This conclusion raises (at least) two questions. Firstly, one may wonder whether the size of a market is a compelling argument to refrain from adopting horizontally consistent investor protection requirements. Even if the answer to this question were in the affirmative, the question arises how the Commission defines a ‘niche market’ as arguably ELTIFs are an equally niche market segment as EuVECA and EuSEF. While there is no official data available on the amount of assets held by ELTIFs, EuVECA or EuSEF, the ESMA public registers provide an overview of the number of ELTIFs, EuVECA and EuSEF. As of November 2020, the ESMA registers indicated that 28 ELTIFs, 13 EuSEF and 387 EuVECA exist.^133^ Hence, at least in terms of the number of funds, the ELTIF market would in fact be even more ‘niche’ than EuVECA. Compared to the over 34,000 UCITS and 29,000 AIFs,^134^ arguably both the ELTIF and EuVECA/EuSEF markets are to be described as ‘niche markets’. The fact that the Commission deliberately refrained from proposing horizontally consistent investor protection requirements in this area without providing objective reasons or compelling arguments therefore lends further support to the idea that the form-over-substance approach employed by EU legislators is mainly motivated by political considerations rather than investor protection and financial stability risks.

#### National Discretion Concerning Non-EU Cases

With respect to non-EU managers (Portfolio E), as elaborated in Sect. 5.1.2 above, it is important to note that they are not yet subject to the EU authorisation and passporting regime but can be marketed to EU investors only where permitted under national legislation (so-called National Private Placement Regimes). In this context, the AIFMD merely provides for a minimum set of conditions that must be met for a non-EU manager to benefit from the possibility to market its funds to EU investors such as the fact that it is not located in a third country that is listed as a Non-Cooperative Country and Territory by the Financial Action Task Force (FATF).^135^ In fact, the vast majority of Member States allow non-EU AIFMs to market their funds to investors in their Member State and in most cases non-EU managers are not subject to any additional substantial rules but merely to registration or notification requirements.^136^ Hence, this is another area where political preferences prevail over regulatory consistency considerations.

#### Inconsistent Disclosures

Equally, inconsistent regulatory approaches can be identified not only with regard to the availability of a marketing passport but also when looking at the content of marketing disclosures to be made to investors. One would generally assume that the Single Rulebook provides for a consistent set of disclosure requirements across all investment funds and only makes distinctions where this is justified due to the specific asset and product-specific features or the types of investors targeted. However, the disclosure requirements are again solely based on the contrived legal form or label used with the newer AIFMD disclosure requirements being generally more granular on many key aspects compared to the UCITS framework. By way of an example, the AIFMD disclosure requirements are more explicit or granular on (liquidity) risk management,^137^ leverage^138^ and delegated functions.^139^

It is not evident why separate disclosure regimes exist or how the aforementioned differences could be justified.^140^ For managers of EuVECA below 500 million EUR, the pre-investment disclosure obligations set out therein^141^ are even significantly shorter than both the UCITS and AIFMD disclosure regimes. This is particularly worth noting since the EuVECA marketing passport enables marketing to wealthier retail investors (investing more than 100,000 EUR), whereas the AIFMD marketing passport does not encompass marketing to even the wealthiest retail investor.

In this context, it is equally interesting to note that the AIFMD and EuVECA regimes allow for the preferential treatment of investors provided that this is disclosed,^142^ whereas the UCITS Directive does not allow for any preferential treatment. In the absence of explanations provided by the EU legislators in any official document, it is incomprehensible why these differences exist or how they could be justified. Even if one tried to argue that preferential treatment (which constitutes a derogation from the general principle of treating all investors fairly and therefore equally) would be justifiable within investment funds sold to professional investors only, such an argument would quickly fall apart given the significant exposures of retail investors to AIFs referred to above and the fact that the EuVECA marketing passport also covers retail investors. In this respect, the ELTIF Regulation provides for more detailed disclosure requirements^143^ and again deviates from the other rulebooks by requiring that preferential treatment for an ELTIF investor is not permitted where the fund is also marketed to retail investors.^144^

In a similar vein, the content of annual reports is not consistent across the various frameworks and their frequency is not based on actual trading activities or redemption frequencies, but defined in a static manner based again on the contrived legal form or label used which is twice-yearly for UCITS^145^ and yearly for AIFs (including EuVECA), despite the fact that in reality the majority of AIFs offer at least monthly portfolio and investor liquidity (61%), many of those even offering *daily* liquidity (62%).^146^

### Risk Management

#### Liquidity Management

The newer risk management rules in the AIFMD framework are significantly more detailed compared to those set out in the UCITS framework. For instance, the AIFMD includes specific Level 1 obligations on liquidity risk management,^147^ whereas the UCITS Level 1 Directive does not explicitly mention liquidity risk management at all. Moreover, the AIFMD rules are complemented by a specific section on liquidity risk management in the Level 2 Regulation,^148^ whereas the UCITS Level 2 text only makes some high-level references to liquidity risk management obligations.^149^ The same holds true for a variety of other risk management-related requirements. By way of an example, the safeguards against conflicts of interest of the risk management function^150^ and obligations for the assessment, monitoring and review of the risk management systems^151^ in the AIFMD are significantly more granular than those set out in the UCITS framework. Moreover, it is important to note that the AIFMD Level 2 takes the form of a regulation that is directly applicable to all AIFMs throughout the Union, whereas the UCITS Level 2 Commission Directive needed to be transposed into national law which further raises the risks of vertical inconsistencies across the EU Member States.

With respect to stress tests (Table 4), the AIFMD requires managers to carry out stress tests to ensure that the risks associated with each investment position of the AIF and their overall effect on the AIF’s portfolio can be properly identified, measured, managed and monitored on an ongoing basis.^152^ Moreover, managers of AIFs are required to regularly conduct stress tests which enable them to assess and monitor the liquidity risk of their AIFs^153^ and report the results of these stress tests to their supervisors.^154^

**Table 4:** Is the manager obliged to carry out stress tests?

Portfolio A	Portfolio B	Portfolio C	Portfolio D	Portfolio E
(a) If set up as UCITS: ‘Where appropriate’ (b) If set up as AIF: Yes	(a) If set up as EuVECA: No (b) If set up as AIF: Yes	Yes	(a) If set up as UCITS: ‘Where appropriate’ (b) If set up as AIF: Yes	Not subject to EU risk management rules

In contrast, the UCITS Level 1 regime remains silent on any form of stress tests. Despite the absence of a Level 1 provision, the UCITS Level 2 Commission Directive states that periodic stress tests and scenario analyses to address risks arising from potential changes in market conditions that might adversely impact the UCITS and liquidity stress tests shall be carried out ‘where appropriate’.^155^ Even ‘where appropriate’, liquidity stress tests under the UCITS framework are limited to exceptional conditions, whereas the AIFMD stress tests should be carried out under both normal and exceptional liquidity conditions. The AIFMD framework even provides for specific Level 2 requirements on how the stress tests should be conducted,^156^ whereas the UCITS texts remain entirely silent on these important risk management matters as well as on the reporting of the stress testing results to supervisors.

Against this background, ESMA wrote a letter^157^ to the European Commission in August 2020 to recommend legislative amendments in the context of the AIFMD review. The first item of the ESMA letter relates to the need to ensure greater regulatory consistency between the AIFMD and UCITS frameworks. In this context, ESMA pointed specifically also to regulatory inconsistencies in the area of liquidity risk management and the liquidity issues faced by some UCITS. ESMA therefore suggested that the European Commission should use the AIFMD review as an opportunity to ensure greater regulatory consistency between the two frameworks.

#### Different Approaches to the Calculation of Leverage

Another risk management-related area where significant regulatory differences can be identified relates to the use and measurement of leverage (Table 5). The AIFMD acknowledges that the use of leverage poses financial stability risks^158^ and therefore defines leverage^159^ and requires that AIFMs implement a leverage limit taking into account certain criteria set out in the AIFMD.^160^ The AIFMD requires detailed leverage-related disclosures to investors^161^ and managers have to report regularly to competent authorities on the use of leverage and shall demonstrate that the leverage limits set by them for each AIF they manage are reasonable and comply with those limits at all times (this will be described further in Sect. 5.4 below). To this end, the leverage calculation methods are further specified in the AIFMD Level 2 provisions which provide detailed rules on the calculation under the gross and commitment methods.^162^

**Table 5:** What is the calculation method for leverage?

Portfolio A	Portfolio B	Portfolio C	Portfolio D	Portfolio E
(a) If set up as UCITS: Subject to national legislation, the following methods *may* be permitted: (i) commitment method (ii) Value-at-Risk (iii) ‘other advanced risk measurement methodologies as may be appropriate’ b) If set up as AIF: (i) gross method (ii) commitment method as both specified in EU legislation	(a) If set up as EuVECA: Not allowed to employ leverage (b) If set up as AIF: (i) gross method (ii) commitment method as both specified in EU legislation	(i) gross method (ii) commitment method as both specified in EU legislation	(a) If set up as UCITS: Subject to national legislation, the following methods *may* be permitted: (i) commitment method (ii) Value-at-Risk (iii) ‘other advanced risk measurement methodologies as may be appropriate’ (b) If set up as AIF: (i) gross method (ii) commitment method as both specified in EU legislation	Not subject to EU risk management rules

Conversely, the UCITS Directive only includes a single reference to leverage^163^ which remains undefined in the UCITS Directive. In addition, the UCITS Directive uses the notion of ‘global exposure’ which equally lacks a definition or further specifications in the UCITS Directive itself. Even the Level 2 requirements on the calculation of UCITS global exposure include only a couple of references to leverage without providing any further clarifications as to the precise definition of leverage or the calculation thereof.^164^ Instead, the UCITS Level 2 provisions provide Member States with discretion to allow for the calculation of global exposure for the application of the commitment approach, the Value at Risk (VaR) approach or ‘other advanced risk measurement methodologies as may be appropriate’. Hence, the UCITS framework simply leaves the important question of the permissible calculation methods for ‘global exposure’ to national legislation which raises the risks of vertical inconsistencies. Clarifications and rules on the calculation of global exposure can, however, be found in the 2010 Guidelines published by ESMA’s predecessor, the Committee of European Securities Regulators (CESR).^165^ In this context, it is worth noting that the CESR Guidelines are not legally binding. Even if they were upgraded in the future to ESMA Guidelines, they would only be subject to a mere comply-or-explain mechanism.^166^

Besides these important legal differences, it is interesting to note that even in terms of substance, the AIFMD leverage regime and UCITS global exposure requirements as specified in the CESR Guidelines follow different approaches on the accepted calculation methods, in particular concerning the use of the VaR model. This is because the CESR Guidelines allow for the use of VaR, although they acknowledge that the VaR approach is a measure of the maximum potential loss due to market risk rather than leverage.^167^ Therefore, the CESR Guidelines acknowledge that ‘there is a risk that the use of the VaR method could result in UCITS strategies using high levels of leverage’^168^ and therefore prescribe safeguards where the VaR approach is used such as ‘regular monitoring of leverage’ and additional disclosures to investors.^169^

In sum, the UCITS and AIFMD frameworks provide for different leverage calculation methods. While it would go beyond the scope of this article to study the adequacy of the VaR approach which has been subject to much criticism from risk experts over the past decades^170^ and has even been described as ‘pure intellectual fraud allowing to take more risks in the fat tails’ by Nassim Taleb,^171^ it is important to note that the European Commission and ESMA expressly rejected the idea of introducing VaR into the AIFMD framework. This was explained, *inter alia*, by the fact that the VaR approach uses correlations which have the ‘propensity to break down in stressed market conditions’.^172^ Interestingly, financial industry associations have since increased their lobbying efforts *vis-à-vis* policy makers to ‘enhance *consistent* measuring and monitoring of leverage in investment funds’. However, their call for consistency does not request that the UCITS rules should be aligned with the AIFMD, but the other way around, namely that the AIFMD framework should be amended to also allow for the use of VaR as is the case for UCITS.^173^

Paradoxically, the regulatory inconsistencies on leverage also contradict the arguments raised by the European Commission that UCITS are less complex and ‘risky’ and are therefore ‘suitable’ products for retail investors, whereas AIFs would lack ‘suitability’ for retail investors, *inter alia*, due to higher leverage. Given the acceptance of VaR, it is not rare to observe UCITS with several hundred^174^ or even several thousand percent leverage.^175^ Meanwhile, only 15% of AIFs are actually reported to be leveraged at all and the majority of those, with the notable exception of hedge funds, make limited use of leverage.^176^ It is therefore not surprising that many of the UCITS facing liquidity problems in recent years reportedly also made more extensive use of leverage.^177^ Just in the 11-month period between July 2018 and June 2019, 16 UCITS managed by three UCITS managers experienced significant investor outflows after a deterioration in portfolio liquidity. In these cases, poor past performance in combination with illiquid asset holdings prompted investors to withdraw their money, which resulted in almost bank-like runs.^178^ A total of 14 of the 16 funds involved were leveraged UCITS funds using the VaR approach.^179^

The recent liquidity shortcomings of some UCITS triggered a heated debate about the adequacy of the UCITS regulatory framework. In this context, the Bank of England Governor Mark Carney even warned of financial stability risks and further stated that ‘These funds [UCITS] are built on a lie, which is that you can have daily liquidity for assets that fundamentally aren’t liquid. And that leads to an expectation of individuals that it’s not that different to having money in a bank.’^180^ The subsequent stress period during the COVID-19 pandemic further highlighted the vulnerabilities of UCITS to liquidity shocks with over 117 UCITS with 54 billion EUR NAV still suspended in September 2020 (despite the fact that the main financial indices had already largely recovered the losses incurred during the previous months).^181^

#### Leverage Ban for Venture Capital

With regard to investments in unlisted securities issued by start-up companies (Portfolio B), it is additionally worth noting that despite the inherent risks involved in venture capital investments, the EuVECA Regulation does not provide for any specific risk management rules apart from the general requirements on conflicts of interest.^182^ On the other hand, unlike the AIFMD and UCITS Directive, the EuVECA Regulation does not allow for the use of leverage at all. The recitals explain that the leverage ban is to ensure that EuVECA do not contribute to systemic risk.^183^

From a (logical) consistency perspective, it remains unclear why leverage in the relatively small EuVECA sector,^184^ which is negligible in size when compared to the AIF and UCITS market (less than a few basis points), would pose systemic risks that needed to be addressed through a regulatory leverage ban whereas the large-scale leverage used by hedge fund AIFs and alternative UCITS or Newcits would not.

Paradoxically, the European Commission’s Impact Assessment includes the statement that ‘data shows that venture capital funds make limited use of leverage’ without providing any further details, references or explanations.^185^ Hence, this even suggests that the leverage ban introduced in the EuVECA Regulation rather addresses an imaginary or theoretical risk that has never really existed in the relevant market.

#### Protecting Retail Investors from ‘Improper Risks’

With respect to infrastructure and other long-term investments (Portfolio C), it is worth noting that, unlike the EuVECA Regulation, only EU managers *authorised* under the AIFMD may make use of the ELTIF label,^186^ meaning that all ELTIFs must be managed in accordance with the AIFMD risk management requirements.^187^ However, unlike other AIFs, ELTIFs are prevented from creating significant synthetic^188^ or financial leverage, *inter alia*, by limiting borrowings to a maximum of 30%.^189^

The need to *limit* ELTIF leverage was explained by the European Commission with the fact that ELTIFs should be prevented from taking ‘improper risks’ given the fact that they are marketed to *retail* investors.^190^ The fact that the Commission considered leverage above 30% as an improper risk for retail investors raises a host of consistency issues, in particular bearing in mind that (i) UCITS can be leveraged way beyond 30% and marketed to retail investors via an EU passport as elaborated in Sect. 5.3.2, (ii) the significant exposures of retail investors to AIFs (other than ELTIFs) referred to in Sect. 5.2.1 where there are no leverage restrictions, and (iii) the outright leverage ban for EuVECA as discussed in Sect. 5.3.4. Consequently, the EU legislation treats the question of whether and to which extent retail investors should be exposed to leveraged investment portfolios in a largely inconsistent way.

#### Non-EU Managers Untroubled by EU Risk Management Requirements

With regard to non-EU managers (Portfolio E), the list of minimum requirements laid down in the AIFMD^191^ that apply to non-EU managers marketing AIFs to EU investors does not cover the risk management provisions. Since the majority of Member States allow non-EU managers to market AIFs in their jurisdictions without additional requirements beyond the minimum list set out in the AIFMD as further elaborated in the sections above, this regulatory inconsistency creates an unlevel playing field between EU and non-EU managers which appears unjustifiable from an investor protection perspective.

From a financial stability point of view, it appears equally illogical not to prescribe (consistent) risk management standards for all AIFs marketed to EU investors, irrespective of the location of the manager of the fund, especially bearing in mind that these marketing activities amount to the NAV of 1.7 trillion EUR and a reported regulatory AuM of 10.3 trillion EUR.^192^

As a result of the unsatisfactory situation described in the sub-sections above, some Member States recently expressed the need to ensure consistency concerning the rules applicable to AIFs marketed by non-EU managers and between the leverage rules set out in the UCITS and AIFMD frameworks.^193^

### Supervisory Powers and Responsibilities

#### Supervisory Powers and Responsibilities in Cross-border Cases

The UCITS V revision addressed a range of inconsistencies between the older UCITS regime and the newer AIFMD provisions with respect to administrative powers and sanctions when carrying out the supervision of investment funds.^194^ However, the UCITS, AIFMD and EuVECA regimes still differ significantly with respect to the competencies in cross-border management cases as the latter two assign almost all supervisory responsibilities and powers to the national competent authority (NCA) of the Member State in which the manager is established.^195^ In contrast, the UCITS Directive contains a delicate split of responsibilities between the NCA where the fund is established and the NCA where the manager is established.^196^

By way of an example, in the case of a suspension of redemptions due to exceptional circumstances (as witnessed most recently due to the liquidity and valuation problems faced by many UCITS and AIFs during the COVID-19 crisis^197^), the UCITS framework requires the fund to inform without delay the home authority of the Member State where it is established as well as the authorities of the Member States where it is marketed of its decision to temporarily suspend the redemption rights of its investors.^198^ Moreover, the powers to require fund suspensions lies with the home NCA of the fund.^199^ In that case, it shall communicate its decision without delay to the host NCAs where the fund is marketed and, in the case of cross-border management, to the home NCA of the manager.^200^

In contrast, the AIFMD remains entirely silent on any obligations for either funds or managers to inform their home or host authorities of the suspension. Similar to the UCITS framework, the AIFMD requires that national authorities shall have the powers to impose a temporary suspension where this is in the interest of investors or the public.^201^ However, unlike the UCITS Directive, it is not the home authority of the fund that decides on this, but the home authority of the manager.^202^ Notwithstanding this, the AIFMD provides for requirements that would under certain circumstances allow the authorities in the Member States where the fund is marketed or managed to impose suspensions.^203^ In case of disagreements, NCAs may bring the matter to the attention of ESMA for a mediation case.^204^

In light of these significant inconsistencies, and given the systemic risk implications of fund suspensions in particular, the European Systemic Risk Board (ESRB) made recommendations to the European Commission in 2017 to clarify the roles and obligations of NCAs and the cooperation between them in cross-border cases.^205^ In 2020, ESMA also requested further legislative clarifications in this area coupled with the general recommendation to ensure greater regulatory consistency between the UCITS and AIFMD regimes.^206^

#### Collection and Exchange of Macro-prudential Data

Regulatory inconsistencies do not only exist with respect to national supervisory competencies and powers but also regarding access to and the exchange of macro-prudential data (i) amongst national authorities and (ii) between national and European authorities. In this regard, the AIFMD provides for a detailed reporting regime whereby AIFMs have to report, on a regular basis, detailed portfolio and risk data to their NCA, including data on leverage. In its Impact Assessment, the European Commission had explained the need for the introduction of such a reporting regime by stating that ‘the absence of a *consistent* approach to the collection of macro-prudential data (on leverage, risk concentrations etc.) and of effective mechanisms for the sharing of this information between prudential authorities at the European or global level is a significant barrier to robust macro-prudential oversight’.^207^ The Commission therefore suggested the introduction of the AIFMD reporting regime ‘to enable the effective monitoring of systemic risks’.^208^ The systemic risk rationale is also laid down in the recitals of the AIFMD^209^ data and, for this reason, the AIFMD also sets out that the data gathered by NCA is to be shared both with other NCAs as well as ESMA and the ESRB.^210^

However, the rationale that the collection of macro-prudential data is a prerequisite for supervisors to identify and address systemic risks is only applied with respect to AIFs (including EuVECA and ELTIF). Conversely, UCITS are not subject to systemic risk reporting requirements (Table 6). This regulatory inconsistency is astounding, especially when bearing in mind that the UCITS account for approximately 60% of the overall net assets in the 17.1 trillion EUR investment fund market in the EU.^211^ In other words: if the EU legislator is convinced that consistent financial and risk data reporting to national and European supervisory authorities is necessary in order to identify and address systemic risks, why would it turn a blind eye to 60% of the market? This inconsistency was pointed out by the ESRB which recommended in 2017 that the European Commission should ‘harmonise’ the UCITS and AIFMD reporting requirements in order to ‘allow for sufficient monitoring of potential vulnerabilities that may contribute to systemic risk’.^212^ In 2020, ESMA reiterated this point and underlined its importance.^213^

**Table 6:** Is the portfolio subject to systemic risk reporting?

Portfolio A	Portfolio B	Portfolio C	Portfolio D	Portfolio E
(a) If set up as UCITS: No (b) If set up as AIF: Yes	Yes	Yes	(a) If set up as UCITS: No (b) If set up as AIF: Yes	Yes

In this context, it is also worth noting that the AIFMD includes explicit obligations for national authorities to exchange with other NCAs as well as with ESMA and the ESRB information relevant for monitoring and responding to the potential implications of the activities of *individual* AIFMs or AIFMs *collectively* for the stability of systemically relevant financial institutions and the orderly functioning of markets, whereas the UCITS framework does not include such provisions.^214^

#### Exchange of Supervisory Information

Beyond this, the supervisory regimes also differ with respect to the information made available to ESMA on day-to-day supervisory matters. By way of an example, the AIFMD obliges NCAs to inform ESMA alongside the other NCAs where they have clear and demonstrable grounds to suspect that acts contrary to this Directive are being or have been carried out by an AIFM not subject to supervision by their own authority,^215^ whereas no such obligation exists in the UCITS framework.

The EuVECA regime even goes a step further than AIFMD by assigning a central role to ESMA to ensure consistent and effective supervision. In particular, NCAs have to share with ESMA the information on the basis of which they granted individual EuVECA licences^216^ and ESMA is explicitly obliged to organise and conduct peer reviews in order to strengthen the *consistency* of national registration processes^217^ as well as peer reviews to strengthen the *consistency* of national processes in relation to supervisory and investigatory powers carried out by competent authorities.^218^ In addition, NCAs are required to inform ESMA without delay where they have clear and demonstrable grounds for believing that the EuVECA managers have committed regulatory breaches and ESMA is explicitly empowered to issue recommendations addressed to the NCAs to take enforcement action or to refrain from doing so.^219^

Therefore, the UCITS, AIFMD and EuVECA frameworks provide for three rather different supervisory regimes and models without any objective reason or justification for the differences and thereby adding more complexity to an already complex EU regulatory and supervisory framework.

#### Leverage Risk Monitoring

Another striking difference between the supervisory powers and responsibilities set out in the UCITS and AIFMD frameworks relates to leverage risk monitoring (Table 7). This is because NCAs are obliged to use the information reported by AIFMs for the purposes of identifying the extent to which the use of leverage contributes to the build-up of systemic risk in the financial system, to risks of disorderly markets or to risks to the long-term growth of the economy.^220^ Where deemed necessary in order to ensure the stability and integrity of the financial system and after having notified ESMA and the ESRB, NCAs shall impose limits on the level of leverage that an AIFM is entitled to employ. The legal text explicitly emphasises that the involvement of ESMA is to ensure that a *consistent* approach is taken by NCAs.^221^ Moreover, ESMA is empowered to determine, taking into account any advice from the ESRB, that the leverage employed by an individual AIFM, or by a group of AIFMs, poses a substantial risk to the stability and integrity of the financial system and to issue advice to NCAs specifying the remedial measures to be taken, including leverage limits.^222^

**Table 7:** Is the portfolio subject to a leverage monitoring regime?

Portfolio A	Portfolio B	Portfolio C	Portfolio D	Portfolio E
(a) If set up as UCITS: No (b) If set up as AIF: Yes	(a) If set up as EuVECA: Not allowed to employ leverage (b) If set up as ordinary AIF: Yes	If set up as AIF: Yes	(a) If set up as UCITS: No (b) If set up as AIF: Yes	Yes

As described above, some UCITS employ high levels of leverage way beyond most AIFs and matched only by highly leveraged hedge fund AIFs. However, while the AIFMD includes the aforementioned supervisory mechanisms to ensure that supervisors monitor and address systemic risks posed by leverage, no such requirements are foreseen in the UCITS framework. Interestingly, the ESRB did not highlight this inconsistency in its 2017 recommendations, but only included a recommendation to ESMA to provide guidance on the leverage monitoring and to limit mechanisms under the AIFMD.^223^

With respect to non-EU managers (Portfolio E), it is also important to note that the AIFMD rules on systemic risk reporting and leverage monitoring by supervisors also cover non-EU managers and non-EU AIFs.^224^ Consequently, an unleveraged plain vanilla US mutual fund or index-tracking US ETF would be subject to a stricter regulatory sporting and supervisory monitoring regime in this respect than an alternative UCITS or Newcits employing hedge fund strategies with complex derivatives and high amounts of leverage.

## Conclusions

The analysis of how four key issues are addressed in the EU investment management legislation reveals significant negative unintended consequences of the form-over-substance approach. In particular, the post-crisis legislative approach is detrimental to achieving regulatory consistency despite the increased importance assigned to the principle of consistency in EU law since the Maastricht and Lisbon Treaties. The idea that one could create a truly consistent regulatory framework by employing a complex patchwork approach with a multitude of separate directives and regulations that need to be individually negotiated, adopted and implemented by a variety of EU and national institutions and then again revised and readjusted to ensure consistency with the other evolving pieces of regulation, has proven to be inadequate to accomplish the desired outcome. Ironically, the s*ingleness* of the EU Single Rulebook is a key component without which it will be difficult, if not practically impossible, to achieve the desired policy goal.

A true Single Rulebook would require working on the premise that all market participants performing similar financial services and all similar financial products are treated equally by EU law, unless there is an objective reason for unequal legal treatment. In this context, any regulatory divide should be duly justified from the viewpoint of the actual risks posed for investor protection and financial stability and not be merely based on the contrived legal form and unsubstantiated assumptions or political preferences. In doing so, EU financial legislation would need to move away from the form-over-substance approach currently being employed, which is based on the creation of artificial legal forms and labels (e.g. AIF, UCITS, EuVECA, ELTIF) which are not accurate indicators of actual investor protection or financial stability risk. Moreover, the magnitude of the problem is growing as in just one decade since the financial crisis, the EU rulebooks regulating the investment management area have gone from one (UCITS Directive) to almost a dozen individual Level 1 directives and regulations regulating fund managers and/or funds, each complemented with numerous Level 2 (and Level 3) acts and thus adding to the overall complexity.

In addition, the EU Single Rulebook currently refrains from regulating many core questions as seen above and instead simply leaves them to national legislation. This approach of combining a multitude of EU rulebooks (instead of a *single* rulebook) with a variety of different national rulebooks with large-scale national discretion on key issues has not only significantly increased the complexity of the overall regulatory framework for investors, financial market participants and supervisors but also constitutes an additional practical barrier to achieving consistent regulatory and supervisory outcomes across the EU Member States.

In light of the findings of this analysis, a more pragmatic alternative legislative approach to achieving a true Single Rulebook would be to follow a risk-based substance-over-form approach to financial regulation. This would require the key rules to be codified in a single EU regulation (that is directly applicable in all EU Member States) and relies on actual risk indicators (e.g. the use of certain complex derivatives, high leverage, illiquid assets) based on concrete and, where possible, quantitative parameters and product features that trigger additional regulatory requirements to mitigate those specific risks. Consistency in financial regulation therefore requires a legislative framework that is non-discriminatory in the sense that it treats risks that are similar in all relevant aspects alike, regardless of the labels or contrived legal forms used. Otherwise, regulatory consistency in the area of EU financial regulation is in danger of becoming a mere buzzword instead of the constitutional principle that it ought to be.

## Footnotes

1 De Larosière et al. (2009); G20, Declaration of the Summit on Financial Markets and the World Economy, available at: http://www.g20.utoronto.ca/2008/2008declaration1115.html. Accessed 28 October 2020.

2 Derossi et al. (2011); Beythan and Virard-Canto (2012); Muller and Zanetti (2012); Johnston (2015); Rehahn (2016); Nabilou (2017a, b); Thomadakis (2017); Hahn (2020).

3 European Fund and Asset Management Association (EFAMA), Quarterly Statistical Report Q2 2020, available at: https://www.efama.org/Publications/Statistics/Quarterly/Quarterly%20Statistical%20Reports/20%2009%20Quarterly%20Statistical%20Release%20Q2%202020.pdf. Accessed 28 October 2020.

4 Eurostat, gross domestic product (GDP) at market prices, available at: https://ec.europa.eu/eurostat/tgm/refreshTableAction.do?tab=table&plugin=1&pcode=tec00001&language. Accessed 28 October 2020.

5 European Securities and Markets Authority (ESMA), Annual Statistical Report EU Alternative Investment Funds 2020, p 30, available at: https://www.esma.europa.eu/file/54227/download?token=JIrB9PSh. Accessed 28 October 2020.

6 Substance-over-form is a long-lasting principle in national and international accounting and tax laws which requires that the economic substance of transactions and events should be considered rather than just their legal form in order to present a true and fair view of the affairs of the entity, see Meyer (1976). In this context, Baker and Hayes (2004) argued that the Enron scandal could have been potentially avoided if the substance-over-form approach had been applied.

7 Duke (1999), p 7.

8 Art. 5 of the Treaty establishing the European Economic Community (EEC).

9 Art. 30(5) of the Single European Act (SEA).

10 Franklin (2011), p 44.

11 First sub-paragraph of Art. C of the TEU.

12 Tietje (1997), p 214.

13 First sentence of the second sub-paragraph of Art. C of the TEU.

14 Second sentence of the second sub-paragraph of Art. C of the TEU.

15 Herlin-Karnell and Konstadinides (2013), p 141.

16 Art. 13 of the TEU.

17 Art. 7 of the TFEU.

18 Franklin (2011), p 59.

19 The Treaties provide in particular a legal framework for the consistency of EU external action with various general principles and objectives as well as provisions specifying institutional responsibilities for consistency, see Nuttall (2005).

20 Second sub-paragraph of Art. 16(6) of the TEU.

21 Art. 17(6)(b) of the TEU.

22 Van Elsuwege and Merket (2012); Hillion (2014).

23 Art. 256(2) of the TEU.

24 Art. 256(3) of the TEU.

25 Art. 62 of the TEU.

26 In its judgment of 6 December 2007 in the case of *Beian v. Romania Beian v. Romania (no. 1)* (no. 30658/05, ECHR 2007-XIII) the Court stated that it is precisely the role of a supreme court to resolve inconsistent case law.

27 Decision of the Court of Chancery delivered on 28 July 1818: *Gee v. Pritchard and Anderson* (1818) 2 Swanston 402 and 414.

28 See the overview provided by van Kampen (2019).

29 Ibid.

30 Ibid.

31 Festinger (1957).

32 Ibid.

33 Mojzisch et al. (2014).

34 ‘Credibility requires consistency’, President of the European Council Herman Van Rompuy, Europe on the World Stage, speech delivered on 31 May 2012 at Chatham House in London, EUCO 107/12, available at: https://ec.europa.eu/commission/presscorner/detail/en/PRES_12_237. Accessed 28 October 2020.

35 Marangoni and Raube (2014).

36 Arts. 13(1), 17(6)(b) and 26(2) of the TEU.

37 Konstadinides (2017).

38 Other languages such as Dutch (‘samenhang’), Danish (‘sammenhæng’) and Swedish (‘samstämmigheten’) use a notion that is best translated into English as ‘connection’.

39 Hoebink (2004); Nuttall (2005); Picciotto (2005); Carbone (2008).

40 Nuttall (2005), p 93

41 Tietje (1997); Duke (1999); Hillion (2008); Stroß (2014); Marangoni and Vanhoonacker (2015).

42 Case C-266/03 *Commission v. Luxembourg* [2005] ECR I-4805; C-433/03, *Commission v. Germany* [2005] ECR I-6985.

43 Organisation for Economic Co-Operation and Development (OECD), The DAC Guidelines—Poverty Reduction—International Development, 2001, p 9, available at: https://www.oecd-ilibrary.org/docserver/9789264194779-en.pdf?expires=1588499076&id=id&accname=guest&checksum=2AFEF849EA7E9E25102C4284D2A81A3B. Accessed 28 October 2020. Interestingly, the OECD also understands policy coherence as a broader notion than consistency involving the systematic promotion of mutually reinforcing policy actions across government departments and agencies and creating synergies towards achieving the defined objective.

44 Christiansen (2001); Nuttall (2005); Stroß (2014); Marangoni and Vanhoonacker (2015).

45 G20, 2008, resolution no. 9, available at: http://www.g20.utoronto.ca/2008/2008declaration1115.html. Accessed 28 October 2020.

46 G20, 2019, commitment 39, available at: http://www.g20.utoronto.ca/analysis/commitments-09-pittsburgh.html. Accessed 28 October 2020.

47 De Larosière et al. (2009).

48 And numerous references to ‘coherence’. The interchangeable use of ‘consistency’ and ‘coherence’ in this report may provide further evidence that the academic debate about the potential different meanings of those terms may not play a significant role in practice. In fact, the report even makes frequent use of the notions ‘coordinated’ and ‘convergent’ as additional linguistic substitutes to consistency and coherence.

49 European Commission Impact Assessment on legislative proposals establishing the European Supervisory Authorities, SEC(2009)1234, p 8. In this context, it is interesting to note that the Commission’s Impact Assessment made several dozen references to ‘consistent’, ‘coherent’, ‘coordinated’ and ‘harmonised’ rules or actions, using these notions in a seemingly interchangeable manner. The same holds true when analysing reports from the European Parliament. By way of an example, the 2015 EP report on stocktaking and challenges of the EU Financial Regulation (2015/2106(INI)) where these notions are equally used in an interchangeable manner.

50 European Banking Authority (EBA): ‘[The Single Rulebook] will close regulatory loopholes and will thus contribute to a more *effective* functioning of the Single Market.’ Retrieved from: https://eba.europa.eu/regulation-and-policy/single-rulebook [emphasis added]. Accessed 28 October 2020.

51 EBA: ‘A more *resilient* European banking sector: A Single Rulebook ensures that prudential safeguards are, wherever possible, applied across the EU and not limited to individual Member States as the crisis highlighted the extent to which Member States’ economies are interconnected.’ [emphasis added]

52 EBA: ‘A Single Rulebook ensures that institutions’ financial situation is more *transparent* and comparable across the EU for supervisors, deposit-holders and investors.’ [emphasis added]

53 EBA: ‘A more *efficient* European banking sector: A Single Rulebook will ensure that institutions do not have to comply with 28 differing sets of rules.’ [emphasis added]

54 Directive 2014/65/EU of the European Parliament and of the Council of 15 May 2014 on markets in financial instruments and amending Directive 2002/92/EC and Directive 2011/61/EU [2014], OJ L 173/349–496.

55 Butler et al. (2018).

56 Babis (2014); Singh (2015); Spendzharova (2016); Wissink (2017).

57 Nabilou (2017a), p 94.

58 EFAMA Quarterly Statistical Report Q2 2020, *supra* n. 3, p 8.

59 European Commission Proposal for a Directive on Alternative Investment Fund Managers, COM(2009) 207 final.

60 Directive 2009/65/EC of the European Parliament and of the Council of 13 July 2009 on the coordination of laws, regulations and administrative provisions relating to undertakings for collective investment in transferable securities [2009] OJ L 302/32–96.

61 Directive 2011/61/EU of the European Parliament and of the Council of 8 June 2011 on Alternative Investment Fund Managers and amending Directives 2003/41/EC and 2009/65/EC and Regulations (EC) No. 1060/2009 and (EU) No. 1095/2010 [2011] OJ L 174/1-73.

62 Regulation (EU) No. 345/2013 of the European Parliament and of the Council of 17 April 2013 on European venture capital funds [2013] OJ L 115/1–17.

63 Regulation (EU) No. 346/2013 of the European Parliament and of the Council of 17 April 2013 on European social entrepreneurship funds [2013] OJ L 115/18–38.

64 Regulation (EU) 2015/760 of the European Parliament and of the Council of 29 April 2015 on European long-term investment funds [2015] OJ L 123/98–121.

65 Regulation (EU) 2017/1131 of the European Parliament and of the Council of 14 June 2017 on money market funds [2017] OJ L 169/8–45.

66 Although the latter is strictly speaking a notion that might be better reserved for certain US funds since it is not used in EU legislation.

67 ESMA Annual Statistical Report, *supra* n. 5, pp 47 et seq.

68 Chapter VII of the UCITS Directive in conjunction with Commission Directive 2007/16/EC (Eligible Assets Directive). However, it is worth noting that UCITS may invest in unlisted securities in accordance with Art. 50(1)(a) of the UCITS Directive up to a limit of 10%. In addition, Art. 50(d) allows for investments (beyond 10%) in recently issued transferable securities, provided that their listing is secured within a year of their issue. As a result of this, the UCITS Directive would not permit large-scale investments in non-listed securities on a permanent basis.

69 Although the definition of what a hedge fund is remains problematic, see Nabilou (2017b).

70 See further Derossi et al. (2011).

71 Art. 50(1)(g) of the UCITS Directive.

72 For example, by using certain over-the-counter (OTC) derivatives or contracts for differences (CFDs) to replicate short exposures synthetically, see further HSCB, A Guide to Alternative UCITS, 2018, available at: https://www.gbm.hsbc.com/-/media/gbm/reports/insights/a-guide-to-alternative-ucits.pdf. Accessed 28 October 2020.

73 At Level 1, the European Parliament and Council adopt the basic laws proposed by the European Commission in the co-decision procedure. At Level 2, the Commission may adopt, adapt and update technical implementing measures, often with the help of consultative bodies such as the European Supervisory Authorities. Level 2 regulatory measures represent an elaboration of the Level 1 text from a technical or implementation perspective, and therefore aim to ensure greater consistency in the interpretation and application of the new legislation among the different Member States. However, developing policy or strategic choices in regulatory and implementing technical standards is not allowed. Further information is available at: https://ec.europa.eu/info/business-economy-euro/banking-and-finance/regulatory-process-financial-services/regulatory-process-financial-services_en#:~:text=At%20level%201%20the%20European,the%20traditional%20co%2Ddecision%20procedure.&text=At%20level%202%20the%20Commission,mainly%20of%20EU%20countries%20representatives. Accessed 28 October 2020.

74 Art. 20 of the AIFMD.

75 Art. 13 of the UCITS Directive.

76 Although some differences remain. By way of an example, the UCITS Level 1 Directive does not explicitly mention the possibility to sub-delegate functions (although commonly used in the UCITS sector), whereas Art. 20(4) to (6) AIFMD specifically address this issue. Another example is that Art. 20(1) of the AIFMD states that delegation arrangements must be notified to the national competent authorities ‘before the delegation arrangements become effective’, whereas Art. 13(1)(a) of the UCITS Directive merely requires that this should be done ‘without delay’.

77 Arts. 75 to 82 of Commission Delegated Regulation (EU) No. 231/2013.

78 Some regulators have therefore asked for future regulation to address these inconsistencies, see e.g. the French Autorité des marchés financiers (AMF), EU2024: Shaping EU27 Capital Markets to meet tomorrow’s challenges, 2019, available at: https://www.amf-france.org/en/news-publications/news/eu2024-shaping-eu27-capital-markets-meet-tomorrows-challenges-focus-areas-and-initial-proposals. Accessed 28 October 2020.

79 Directive 2014/91/EU of the European Parliament and of the Council of 23 July 2014 amending Directive 2009/65/EC on the coordination of laws, regulations and administrative provisions relating to undertakings for collective investment in transferable securities (UCITS) as regards depositary functions, remuneration policies and sanctions [2014] OJ L 257/186–213.

80 European Commission Impact Assessment on UCITS V, SWD(2012) 185 final.

81 Directive (EU) 2019/1937 (Whistleblower Directive) will introduce additional rules on whistleblower protection in relation to AIFMs and thereby increase the level of consistency with the UCITS V rules on whistleblowing as from December 2021 or December 2023 depending on the size of the relevant entity, although significant differences will remain between the two whistleblower regimes.

82 Chapter II of the AIFMD.

83 Section 1 of Chapter III of the UCITS Directive.

84 Chapter II of the UCITS Directive.

85 As regards redemption frequencies, Art. 1(2) in conjunction with Art. 76 of the UCITS Directive require that all UCITS offer at least twice-monthly redemption rights which can be reduced to only monthly redemptions subject to the permission of the competent authorities.

86 Chapter VII of the UCITS Directive in conjunction with Commission Directive 2007/16/EC (Eligible Assets Directive).

87 Notable exceptions are ELTIF, EuVECA, EuSEF and MMFs set up as AIFs. However, only the regulation on MMF is mandatory, whereas the other three regulations are merely voluntary.

88 Art. 3(2)(a) of the AIFMD.

89 Art. 3(2)(b) of the AIFMD.

90 See European Commission Questions and Answers on the AIFMD, p 13, available at: https://ec.europa.eu/info/sites/info/files/aifmd-commission-questions-answers_en.pdf. Accessed 28 October 2020.

91 De Larosière et al. (2009), p 29.

92 Gomes et al. (2020).

93 Schiavello and Graziani (2020).

94 The same holds true for EuVECA, EuSEF and ELTIF managers. Hence, the UCITS, EuVECA, EuSEF and ELTIF labels are only accessible to EU managers.

95 Chapter VII of the UCITS Directive.

96 European Commission, 30th meeting of the Expert Group of the European Securities Committee on 27 September 2019, Ares(2019)6840641.

97 Art. 1(1) in conjunction with Arts. 2(e), 5 and 27 of the UCITS Directive.

98 Art. 2(c) in conjunction with Art. 7(1)(d) of the UCITS Directive.

99 Art. 42 of the AIFMD.

100 ESMA’s advice to the European Parliament, the Council and the Commission on the application of the AIFMD passport to non-EU AIFMs and AIFs, 2016, available at: https://www.esma.europa.eu/sites/default/files/library/2016-1140_aifmd_passport.pdf. Accessed 28 October 2020.

101 See the overview provided by the law firm CMS, available at: https://cms.law/en/media/local/cms-cmno/files/publications/guides/cms-guide-to-private-placement-of-funds-2019. Accessed 28 October 2020.

102 ESMA Annual Statistical Report, *supra* n. 5, pp 30-31.

103 Ibid.

104 ESMA Annual Statistical Report, *supra* n. 5, p 20.

105 European Commission Impact Assessment on AIFMD, SEC(2009) 576, pp 6 and 61.

106 ESMA Annual Statistical Report, *supra* n. 5, pp 6 et seq.

107 Art. 43 of the AIFMD.

108 See the overview provided by the law firm CMS available at: https://cms.law/en/media/local/cms-cmno/files/publications/guides/cms-guide-to-passporting-aif-2019. Accessed 28 October 2020.

109 ESMA Annual Statistical Report, *supra* n. 5, p 4. However, the actual retail participation could be significantly higher given the circumstances explained on p 9.

110 Ibid.

111 EFAMA Asset Management Report 2019, p 5, available at: http://www.efama.org/Publications/Statistics/AssetManagement Report/AssetManagementReport2019.pdf. Accessed 28 October 2020.

112 Art. 6(2) of the AIFMD.

113 See further ESMA Report on Trends, Risks and Vulnerabilities No. 2 2020, available at: https://www.esma.europa.eu/sites/default/files/library/esma_50-165-1287_report_on_trends_risks_and_vulnerabilities_no.2_2020.pdf. Accessed 28 October 2020.

114 In this context, it is worth noting that the revised EuVECA Regulation that has been applicable since March 2018 includes an exception to the general rule that EuVECA must be invested in non-listed small and medium-sized enterprises (SMEs), namely in the case where SMEs are listed as an SME growth market. The same holds true for EuSEF which is subject to identical rules on most aspects covered in this article. Given the low relevance of EuSEF in practice (only 13 EuSEF exist as of October 2020 according to the public register on the ESMA webpage), the following sections will focus on EuVECA.

115 Art. 6 of the EuVECA Regulation.

116 European Commission Impact Assessment on a revision of the European Venture Capital Funds Regulation, SWD(2016) 228 final, Annex 10 [emphasis added].

117 Ibid., Annex 9.

118 European Commission Impact Assessment on the European Venture Capital Funds Regulation, SEC(2011) 1515 final, p 54.

119 Ibid.

120 European Commission Impact Assessment on European Venture Capital Funds, *supra* n. 118, pp 5 et seq.

121 European Commission Proposal for a Regulation on European Venture Capital Funds, 2011/0417 (COD), Art. 6.

122 EU Parliament debate on the proposed EuVECA Regulation on 12 September 2012, minutes available at: https://www.europarl.europa.eu/sides/getDoc.do?type=CRE&reference=20120912&secondRef=ITEM-017&language=EN&ring=A7-2012-0193. Accessed 28 October 2020.

123 Council proposals on the EuVECA Regulation, 10826/12, p 6.

124 Chapter V of the ELTIF Regulation.

125 Art. 26 of the ELTIF Regulation.

126 Arts. 27 and 28 of the ELTIF Regulation.

127 Art. 30(3) of the ELTIF Regulation.

128 Art. 30(1) of the ELTIF Regulation.

129 Art. 30(6) of the ELTIF Regulation.

130 European Commission Impact Assessment on ELTIF Regulation, SWD(2013) 230 final, p 33.

131 Ibid., p 34.

132 European Commission Impact Assessment on a revision of the European Venture Capital Funds Regulation, *supra* n. 116, p 33.

133 ESMA public registers, available at: https://www.esma.europa.eu/databases-library/registers-and-data. Accessed 9 September 2020.

134 EFAMA Quarterly Statistical Report Q2 2020, *supra* n. 3, p 8. Accessed 9 September 2020.

135 Art. 42 of the AIFMD.

136 See the overview provided by the law firm CMS, available at: https://cms.law/en/media/local/cms-cmno/files/publications/guides/cms-guide-to-private-placement-of-funds-2019. Accessed 28 October 2020.

137 Art. 23(1)(a) and (h) of the AIFMD for which there is no equivalent in Schedule A of Annex I of the UCITS Directive.

138 Art. 23(1)(a) of the AIFMD. In this context, it is worth noting that ESMA and its predecessor, the Committee of European Securities Regulators (CESR), sought to address this shortcoming by publishing (non-binding) Q&As (ESMA34-43-392—Section V) and CESR Guidelines (CESR/10-788—Section 3.8) that require the disclosures of leverage with regard to UCITS.

139 Art. 23(1)(f) of the AIFMD.

140 Significant differences also continue to exist with respect to key investor documents under the UCITS framework and the Regulation on packaged retail investment and insurance products (PRIIPs). However, it would go beyond the scope of this article to analyse those issues.

141 Art. 13 of the EuVECA Regulation.

142 Art. 7(g) of the EuVECA Regulation.

143 Chapter IV of the ELTIF Regulation.

144 Art. 30(4) of the ELTIF Regulation.

145 Art. 68(1) of the UCITS Directive, Art. 22(1) of the AIFMD, Art. 12(1) of the EuVECA Regulation.

146 ESMA Annual Statistical Report, *supra* n. 5, pp 6 et seq.

147 Art. 16 of the AIFMD.

148 Arts. 46 to 49 of Commission Delegated Regulation (EU) No. 231/2013.

149 Arts. 23(4), 28(1) and 40(3) and (4) of Commission Directive 2010/43/EU.

150 Art. 43 of Commission Delegated Regulation (EU) No. 231/2013.

151 Art. 41 of Commission Delegated Regulation (EU) No. 231/2013.

152 Art. 15(3)(b) of the AIFMD.

153 Second sub-paragraph of Art. 16(1) of the AIFMD.

154 Art. 24(2)(e) of the AIFMD.

155 Second sub-paragraph of Art. 40(3) of Commission Directive 2010/43/EU.

156 Art. 48 of Commission Delegated Regulation (EU) No. 231/2013.

157 ESMA letter to the European Commission on the review of the AIFMD, https://www.esma.europa.eu/sites/default/files/library/esma34-32-551_esma_letter_on_aifmd_review.pdf. Accessed 28 October 2020. In this letter, ESMA asked the European Commission to propose legislative changes to address regulatory inconsistencies between the UCITS Directive and the AIFMD. Moreover, the letter also pointed to the need to ensure greater consistency between UCITS/AIFMD and MiFID II.

158 Recital 49 of the AIFMD.

159 Art. 4(1)(v) of the AIFMD.

160 Art. 15(4) of the AIFMD.

161 Art. 23(1)(a) and (5) of the AIFMD.

162 Arts. 6 to 11 of Commission Delegated Regulation (EU) No. 231/2013.

163 Art. 19(3)(c) of the UCITS Directive.

164 Art. 41 of the UCITS Level 2 Directive.

165 CESR Guidelines on Risk Measurement and the Calculation of Global Exposure and Counterparty Risk for UCITS (CESR/10-788).

166 Art. 16(3) of Regulation (EU) No. 1095/2010. See further van Rijsbergen (2018).

167 Para. 38 of the CESR Guidelines.

168 Para. 75 of the CESR Guidelines.

169 Boxes 23 to 25 of the CESR Guidelines.

170 DerivSource article on ‘VaR Confusion in UCITS Rules Needs Fixing’, 2009, available at: https://derivsource.com/2009/05/19/var-confusion-in-ucits-rules-needs-fixing-says-em-applications. Accessed 28 October 2020. N. Taleb, Against Value-at-Risk: Nassim Taleb Replies to Philippe Jorion, 1997, available at: https://www.fooledbyrandomness.com/jorion.html. Accessed 28 October 2020.

171 The Economist interview with Nassim Taleb, 2012, available at: https://www.economist.com/news/2012/11/21/from-fat-tails-to-fat-tony. Accessed 28 October 2020.

172 ESMA’s technical advice to the European Commission on possible implementing measures of the AIFMD, ESMA/2011/379, p 189.

173 AMIC/EFAMA Joint Paper on the use of leverage in investment funds in Europe, 2017, available at: https://www.efama.org/Publications/Public/170719_AMICEFAMAleveragepaper.pdf [emphasis added]. Accessed 28 October 2020.

174 By way of an example, this UCITS discloses that notional leverage figures ‘may reach high levels’ and that it is not expected to exceed 1500%: https://investment-solutions.mercer.com/content/dam/mercer-subdomains/delegated-solutions/FundSupplementsMGI/MercerAbsoluteReturnFixedIncomeFund.pdf. Accessed 28 October 2020.

175 For example, the following UCITS discloses that ‘based on historical data’, the level of leverage is not expected to exceed 4700%: https://ie.lyxorfunds.com/en/instit/documents/prospectussuppl/en/IE00B4Q7X812?category=document&utm_source=webSite. Accessed 28 October 2020.

176 ESMA Annual Statistical Report, *supra* n. 5, p 6.

177 For example, see Institutional Investor article on ‘Clinton Group’s Quant Meltdown’, 2017, available at: https://www.institutionalinvestor.com/article/b1505phdyg4z5k/clinton-groups-quant-meltdown. Accessed 28 October 2020.

178 L. Vivar, M. Wedow and C. Weistroffer, Is leverage driving procyclical investor flows? Assessing investor behaviour in UCITS bond funds, 2019, available at: https://www.ecb.europa.eu/pub/financial-stability/macroprudential-bulletin/html/ecb.mpbu201910_4~a9c04beceb.en.html—toc1. Accessed 28 October 2020.

179 Ibid.

180 Reuters article ‘Illiquid investment funds “built on a lie”, BoE’s Carney says’, 2019, available at: https://www.reuters.com/article/uk-woodford-inv-suspension-carney/illiquid-investment-funds-built-on-a-lie-boes-carney-says-idUSKCN1TR1LI. Accessed 28 October 2020.

181 See the article by Fitch rating, available at https://www.fitchratings.com/research/fund-asset-managers/h2o-suspensions-show-liquidity-risk-still-stalks-mutual-funds-01-09-2020. Accessed 28 October 2020. Although some UCITS suspensions appear not to have been caused by the pandemic-related market turbulences, but by investments in illiquid assets.

182 Art. 9 of the EuVECA Regulation.

183 Recital 23 of the EuVECA Regulation.

184 The Explanatory Memorandum of the EuVECA Regulation estimates the size of the whole European venture capital sector in the period from 2003 to 2010 at € 64 billion out of a total of € 437 billion invested in the wider field of private equity; this would be less than 15% of the overall private equity market back then and just a few basis points compared to the total size of the AIF and UCITS markets where no such leverage ban exists.

185 European Commission Impact Assessment on a revision of the European Venture Capital Funds Regulation, *supra* n. 116, Annex 10.

186 Art. 5(2) of the ELTIF Regulation.

187 Art. 7(2) of the ELTIF Regulation.

188 Art. 9(2) of the ELTIF Regulation.

189 Art. 16(1) of the ELTIF Regulation.

190 European Commission Impact Assessment on ELTIF Regulation, *supra* n. 130, pp 34 et seq.

191 Art. 42 of the AIFMD.

192 ESMA Annual Statistical Report, *supra* n. 5, p 30.

193 European Commission, 30th meeting of the Expert Group of the European Securities Committee 27 September 2019, Ref. Ares(2019)6840641.

194 Beythan and Virard-Canto (2012); Muller and Zanetti (2012).

195 Arts. 45 and 46 of the AIFMD; Art. 18 of the EuVECA Regulation.

196 Arts. 19, 97 and 108 UCITS Directive.

197 Financial Times article on ‘European regulators ramp up scrutiny of investment fund liquidity’, 2020, available at: https://www.ft.com/content/69e46be8-9513-4f15-ba10-0f9b70ca1c55. Accessed 28 October 2020.

198 Art. 84(3) of the UCITS Directive.

199 Arts. 97(3) and 98(2)(j) of the UCITS Directive in conjunction with Art. 12(1)(b) of Commission Regulation (EU) No. 584/2010.

200 Ibid.

201 Art. 46(2) of the AIFMD.

202 Art. 45 of the AIFMD.

203 Art. 45(6) of the AIFMD.

204 Art. 45(10) of the AIFMD.

205 ESRB recommendations on liquidity and leverage risks in investment funds, ESRB/2017/6, recommendation A(2).

206 ESMA letter to the European Commission, *supra* n. 157.

207 European Commission Impact Assessment on AIFMD, SEC(2009) 576, p 26 [emphasis added].

208 Ibid., p 30.

209 Recital 49 of the AIFMD.

210 Art. 25(2) of the AIFMD.

211 ESMA Annual Statistical Report, *supra* n. 5, p 8.

212 Recommendation D of the European Systemic Risk Board of 7 December 2017 on liquidity and leverage risks in investment funds (ESRB/2017/6).

213 ESMA letter to the European Commission, *supra* n. 157.

214 Art. 53 of the AIFMD.

215 Art. 50(5) of the AIFMD.

216 Art. 16a(1) of the EuVECA Regulation.

217 Art. 14(9) and (10) of the EuVECA Regulation.

218 Art. 19 of the EuVECA Regulation.

219 Art. 21(5) of the EuVECA Regulation.

220 Art. 25(1) of the AIFMD.

221 Art. 25(5) of the AIFMD. Moloney (2011), p 194.

222 Art. 25(7) of the AIFMD.

223 ESRB recommendations, *supra* n. 205, recommendation E.

224 Art. 42 of the AIFMD, although the enforceability of leverage limits *vis-à-vis* non-EU AIFMs might be complicated in the absence of more explicit provisions. It is, however, worth noting that Art. 47(4) of the AIFMD provides ESMA with powers to impose certain restrictions on non-EU AIFMs and even to ban their marketing activities in the Union.
